# Determinants of vitamin D status in young adults: influence of lifestyle, sociodemographic and anthropometric factors

**DOI:** 10.1186/s12889-016-3042-9

**Published:** 2016-05-11

**Authors:** Rune Tønnesen, Peter Hambak Hovind, Lars Thorbjørn Jensen, Peter Schwarz

**Affiliations:** Department of Clinical Physiology and Nuclear Medicine, Rigshospitalet, Nordre Ringvej 57, 2600 Copenhagen, Glostrup Denmark; Department of Endocrinology PE and Research Centre of Ageing and Osteoporosis, Rigshospitalet, Copenhagen, Denmark; Department of Clinical Physiology and Nuclear Medicine, University Hospital of Herlev, Copenhagen, Denmark; Faculty of Health Sciences, University of Copenhagen, Copenhagen, Denmark

**Keywords:** Vitamin D, Young adults, Season, Sex, Education, Smoking, Fast food, Alcohol, Exercise

## Abstract

**Background:**

Very few studies have investigated the determinants of circulating 25-hydroxyvitamin D (25[OH]D) in young adults (18–25 years old) using a set of variables that include lifestyle, sociodemographic, and anthropometric data. Our aim was to investigate the association between these variables and vitamin D status in a sample of untreated young adults.

**Methods:**

A total of 738 young adults were enrolled in a (June cross-sectional study 2012 to May 2014) and were recruited from educational institutions in the Copenhagen area. For multivariate logistic regression subjects was categorized based on 25[OH]D in serum into; vitamin D sufficiency (S-25[OH]D > 50 nmol/L), vitamin D insufficiency (25 nmol/L ≤ S-25[OH]D ≤ 50 nmol/L), vitamin D deficiency (S-25[OH]D < 25 nmol/L). Information on lifestyle factors and education was obtained by self-reported questionnaires.

**Results:**

700 subjects with a valid measurement of S-25[OH]D and a completed questionnaire was analysed. 238 had vitamin D insufficiency, 135 had vitamin D deficiency of which 13 had severe vitamin D deficiency (S-25[OH]D < 12.5 nmol/L). The relative risk (RR) for vitamin D deficiency was highest for men 2.09 (1.52, 2.87); obese subjects 2.00 (1.27, 3.15); smokers 1.33 (1.02, 1.73); subjects who exercised 0-½ hours a week 1.88 (1.21, 2.94); and subjects who consumed fast food once a week 1.59 (1.05, 2.43). The relative risk was significantly lower for subjects who were studying for a Bachelor’s degree (0.40 (0.23, 0.68). For vitamin D insufficiency, the highest RR was again for men 1.31 (1.06, 1.61); obese subjects 1.57 (1.17, 2.11); and subjects who exercised 0-½ hours a week 1.51 (1.11, 2.06).

**Conclusion:**

In this study of young adults, vitamin D deficiency was highly prevalent. Modifiable factors such as smoking, maintenance of normal BMI, and physical activity are all potential targets for interventional trials to determine the causal order; such knowledge would be useful in improving S-25[OH]D in young adults. The small group with severe vitamin D deficiency warrants increased attention.

## Background

Vitamin D in its hydroxylated form, 25-hydroxyvitamin D (25-[OH]D), is considered a reliable marker of vitamin D status [1]. 25-[OH]D is substrate for the dihydroxylated form 1,25-dihydroxyvitamin D (1,25-[OH]D), which is the hormonally active form and necessary for normal bone calcification and remodelling processes [2]. Vitamin D deficiency (S-25[OH]D < 25 nmol/l) leads to secondary hyperparathyroidism, insufficient bone calcification and most likely osteomalacia with an increased risk of osteoporosis, falls and fractures [3–6]. The optimal 25(OH)D concentration required to prevent osteoporosis is disputed [7–9]; for the purpose of this study, we considered a circulating S-25[OH]D concentration above 50 nmol/L to be sufficient, and thus, an S-25[OH]D between 25 and 50 nmol/L is considered insufficient [7].

In humans, bone mass increases with the calcification of bone during childhood and youth. The maximal bone mass, peak bone mass (PBM), is reached the age of 25 and 35 years and is primarily influenced by vitamin d status, calcium intake and physical activity [10]. In males, vitamin D status predicts peak bone mass [11]. However, this is not directly applicable to females [12].

Vitamin D stems primarily from endogenous synthesis in the epidermis, for which UVB-light exposure, and heat are necessary to produce vitamin D_3_ (cholecalciferol) [13]. Diet is the exogenous source of vitamin D; plant-based foods provide vitamin D_2_ (ergocalciferol), and Animal products provide vitamin D_3_.

Seasonal variations of UVB correspond to seasonal fluctuations in the synthesis of vitamin D in the skin, primarily in countries of high latitudes, e.g., Denmark at 56° N. In summer months (15 April -14 October), the primary source of vitamin D is endogenous. In contrast, in winter months (15 October to 14 April), the source of vitamin D is mainly exogenous from food such as fatty fish, including salmon, herring, and mackerel [14].

Vitamin D is synthesized in the liver to the pre-hormone 25[OH]D and subsequently hydroxylated in the kidney to the hormonal active form 1,25-dihydroxyvitamin D3 (1,25[OH]D).

Excessive calorie intake can increase body mass index (BMI), which several studies have found to be inversely associated with vitamin D status [15, 16]. Obesity and BMI are inversely related to socioeconomic status [17], and evidence exists that suggests an association between lower socioeconomic status and a higher risk of diet-related conditions. Of socioeconomic determinants, educational level is the primary driver of a healthy diet [18]; lower educational level is associated with low food involvement and poor diet quality [19]. The association between vitamin d status and education is well established in the adult population aged over 30 years [20].

The association between physical activity and increased vitamin D status is also well established [21, 22]. It is unclear if the association stems from increased sun exposure during outdoor activity as the association is found in the winter as well [23]. Other lifestyle factors, such as alcohol and tobacco use [20, 24], could also play a role in vitamin D status. However, their influence on vitamin d status remains debated [16].

Vitamin D insufficiency and worse has been linked increased risk for development of several cancer types [25], autoimmune diseases like type 1 diabetes and multiple sclerosis and increased risk of metabolic syndrome, hypertension and cardiovascular diseases [25, 26].

If vitamin D deficiency has a role in the development of high blood pressure, heart disease and increases the risk of osteoporosis, then it is important to investigate the extent of vitamin D deficiency. Furthermore, knowledge on sociodemographic and lifestyle topics importance for S-25-[OH]D concentration could aid in health promoting actions in subgroups among young adults. Low S-25-[OH]D is easily corrected by vitamin D supplementation, to potential benefit for the individual and public health.

Over the last 15 years in Denmark, exercise and fish consumption has decreased in the 19-25 age group as compared to past decades [27]. Both changes can negatively affect the accrual of mineral content in bones. Among 35670 subjects aged 15–30 years, who visited a General Practitioner in Copenhagen, 11150 (31,3 %) had vitamin D insufficiency (25 nmol/L ≤ S-25[OH]D < 50 nmol/l) and 9690 (27.2 %) had vitamin D deficiency [28]. The causes for low vitamin d status may be multifactorial.

In most studies, only older populations including subjects with chronic diseases are studied, and only a limited number of factors related to S-25[OH]D are investigated simultaneously [20, 29]; there is a lack of information on the effects of simultaneous factors in young populations. To promote the public health aspect, we needed to investigate multiple predictors together as certain combinations of predictive factors could be protective for low S-25[OH]D. In the present study, we aimed to investigate determinants of vitamin D status in untreated young adults - sociodemographic, lifestyle, and anthropometric factors - to determine which variables are risk factors for vitamin D deficiency and insufficiency.

## Methods

### Study population

**Fig. 1:**
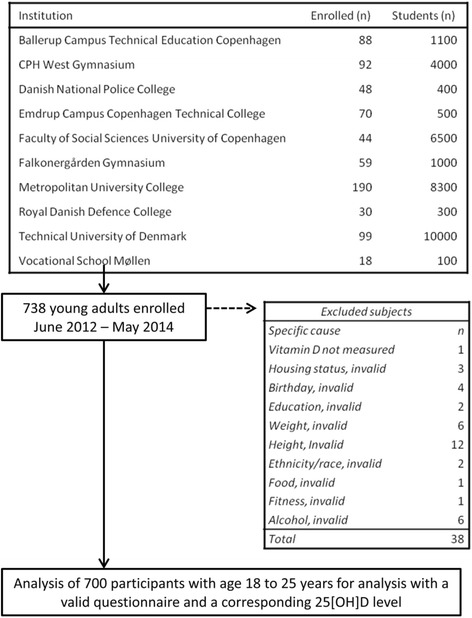
Flowchart for recruitment

The cross-sectional population study was carried out among Copenhagen citizens from June 2012 to May 2014 (Flowchart for recruitment, see Fig. 1). The screening comprised 738 young adults; 38 were excluded due to missing data (height, weight, vitamin D status, and ethnicity). A total of 700 subjects (94,9 %) were subject to analysis including a complete questionnaire and corresponding serum 25[OH]D status assessment. The target study population was young adults. Participants were recruited from a booth set up in the lobbies or hallways at educational institutions in Copenhagen. From the institution’s administrative offices we obtained estimates on number of students; it was impossible to get accurate numbers of students as almost institutions have short term courses. Age distribution was not obtainable from the administrative offices. See Fig. 1 for educational institutions and no. of students. The educational institutions were selected on the basis of their educational programs, to ensure representation from the different educational levels; Vocational/Trade School, Gymnasium, Bachelor’s degree, and Master’s degree. The inclusion criterion was being aged between 18 to 25 years. Exclusion criteria were any known cancers, calcium metabolic diseases, bone disease, ongoing vitamin D supplementation, hypertension, heart disease, pregnancy, diabetes, epilepsy, and use of anabolic/systemic steroids.

### Questionnaire

Apart from date of birth, height (cm), weight (kg), ethnicity, and gender, the following variables were included in the self-reported questionnaire:

- *Exercise habits:* “How many hours have you exercised in the last seven days?”: 0-½, ½-2, 2–4, 4–7, or 7 or more.
- *Smoking:* “How many cigarettes have you smoked in the last seven days?” non-smoker, passive smoker, 1–6, or 7 or more.
- *Alcohol:* “How many units (12 g) of alcohol have you had to drink in the last seven days?”: none, 1–2, 3–4 or 5 or more.
- *Food habits:* “How many times have you ingested fast-food (i.e., pizza, burger, sausages, or shawarma) in the last seven days?”: 0, 1, 2–3, 4–6, 7 or more.
- *Education:* Vocational/Trade School, Gymnasium, Bachelor’s degree, Master’s degree.
- *Status of living:* Live with parents or have moved out

The self-reported values for weight and height were used to compute BMI as the weight in kg divided by height in metres squared (kg/m^2^). BMI was grouped into: underweight; BMI < 18 kg/m^2^, normal weight; 18.5 kg/m^2^ ≤ BMI < 25 kg/m^2^, overweight; 25 ≤ kg/m^2^ BMI < 30 kg/m^2^, obese; BMI ≥ 30 kg/m^2^

Ethnicity, based on the question “origin of the mother”, was divided into two groups; “Caucasian” and “Other”. The “Caucasian” group includes the groups “Europe” and “Middle East” and was equivalent to the U.S. Census 2010 definition of “White”.

Variables were grouped into non-modifiable; season, sex and ethnicity/race and modifiable; exercise, smoking status, fast food, education, living status and BMI.

### Biochemical analysis

All participants provided a non-fasting venous blood sample of 16 mL. The serum was stored at −20 °C until analysis, which took place less than a week after sampling. For analyses of 25[OH]D, we used ChemiLuminescent ImmunoAssay (CLIA Diasorin, Stillwater, Minnesota, USA). The inter-assay coefficient of variation was less than 10 %. The lower detection limit was 10 nmol/L, and the upper detection level was 220 nmol/L. Subjects with S-25[OH]D below 10 nmol/L were assigned the value 10 nmol/L

Based on S-25[OH]D subjects was grouped into three groups [7]: Vitamin D sufficiency; S-[OH]D > 50 nmol/L, vitamin D insufficiency; 25 nmol/L < S-25[OH]D ≤ 50 nmol/L and vitamin D deficiency; S-25[OH]D ≤ 25 nmol/L. The group severe vitamin D deficiency; S-25[OH]D ≤ 12.5 nmol/L is contained in the vitamin D deficiency group as they only consist of 13 subjects.

### Statistical analyses

Power analysis was done one beforehand. The least relevant difference was estimated at 10 nmol/L and standard deviation (SD) = 20 nmol/L. Based on alpha = 0.05 and a nominal power of 0.80, the study required at least 64 participants in each group.

Categorical data were summarized as percentages. Continuous data were summed up as the means and SD when data followed a normal distribution or medians and interquartile range (IQR) when they did not. Visual inspection of the continuous data was used to determine the distribution.

Multivariate robust logistic regression was applied to determine independent predictors of S-25[OH]D concentration below 25 nmol/L and 50 nmol/L, [30]. A means of association between vitamin d status and the explanatory factors, the relative risk (RR) a.k.a. prevalence rate ratio was chosen because low vitamin D status was typical. RR for vitamin D deficiency and insufficiency, both compared to sufficiency was estimated by multivariate-adjusted logistic regression analyses to calculate the association between predictor variables, adjusted for all other predictor variables; season sex and ethnicity/race, exercise, smoking status, fast food, and education, living status and BMI groups. Test for linear relation was performed by the inclusion of the variables as continuous variables in the model and is reported as RR for a one unit with 95 % confidence intervals. Multivariate regressions models were not adjusted for confounders. SAS, version 9.3 (SAS Institute Inc. Cary, NC USA) was used for all statistical analyses.

## Results

In this study, we analysed a convenience sample of 700 community-dwelling subjects. In total, 339 women (mean age 22.0 ± 2.2 years) and 361 men (mean age 21.6 ± 2.3 years) were included in the analysis, primarily recruited in the winter season (Table 1). Serum concentration of 25[OH]D was between 10 nmol/L (lower detection limit) and 211 nmol/L for all participants. Overall, 135 participants had vitamin D deficiency, and 238 had vitamin D insufficiency. Among the total of 135 with vitamin D deficiency, eleven had severe vitamin D deficiency; two subjects (one man and one woman) in the summer both non-Caucasians had severe vitamin D deficiency and in the winter 11 subjects; five women (one Caucasian and four non-Caucasian) and six men (four Caucasians and two non-Caucasians).

**Table 1:** Characteristics of participants

Variables	Women (*n* = 339)	Men (*n* = 361)
Age (year)	22.0 ± 339.0	2.3 ± 0.0
Height (m)	168 ± 339	7.1 ± 0.0
Weight (kg)	63.8 ± 339.0	11.6 ± 0.0
BMI (kg/m^2^)	22.6 ± 339.0	3.0 ± 0.0
S-25[OH]D^a^ (nmol/L)	53 (35 – 69)	40 (25 - 63)
*Season*		
Summer (%)	26.0	25.8
Winter (%)	74.0	74.2
*Ethnicity/Race*		
Caucasian (%)	88.8	91.7
Other (%)	11.2	8.3

Normally distributed data are expressed as the mean +/- SD. ^a^Non-normally distributions are expressed as median (IQR)

### Modifiable variables

Increased time spent exercise was associated with decreased RR for vitamin D deficiency, for a one hour increase in time spent exercise was associated with RR = 0.91 (0.85 - 0.97). The same was true vitamin D insufficiency where for one hour increase in time spent in exercise RR = 0.94 (0.90 - 0.98). Exercise did not interact with season or gender.

Smoking was associated with a higher RR = 1.33 (1.02, 1.73) for vitamin D deficiency compared with non-smoking. There was no association between smoking and vitamin D insufficiency (Table 2).

**Table 2:** Prevalence and multivariate adjusted relative risk (RR) with (95 % Cl) for vitamin D deficiency and insufficiency compared to the sufficient group S-[OH]D > 50 nmol/L

	Vitamin D deficiency			Vitamin D insufficiency		
	S-25[OH]D ≤ 25 nmol/L			25 nmol/L < S-[OH]D ≤ 50 nmol/L		
Modifiable variables	n/n_total_	RR	P	n/n_total_	RR	P
*Exercise (hours last 7 days)*						
7 and above	22/112	1 Reference	.	44/134	1 Reference	.
4 to 7	17/85	0.99 (0.60, 1.64)	0.97	33/101	0.91 (0.64, 1.30)	0.61
2 to 4	24/92	1.12 (0.71, 1.76)	0.64	52/120	1.22 (0.88, 1.69)	0.24
1/2 to 2	38/107	1.55 (1.02, 2.36)	0.04	59/128	1.26 (0.92, 1.72)	0.16
0 to 1/2	34/66	1.88 (1.21, 2.94)	0.01	50/82	1.51 (1.11, 2.06)	0.01
*Smoking status (last 7 days)*						
Non Smoker	82/315	1 Reference	.	152/385	1 Reference	.
Smoker	53/147	1.33 (1.02, 1.73)	0.034	86/180	1.08 (0.88, 1.33)	0.455
*Alcohol (units last 7 days)*						
0 Non drinker	77/214	1 Reference	.	103/240	1 Reference	.
1 to 4	29/102	0.73 (0.52, 1.03)	0.08	54/127	0.99 (0.77, 1.28)	0.94
5 or more	29/146	0.68 (0.47, 0.97)	0.03	81/198	0.99 (0.79, 1.25)	0.94
*Fast food (meals last 7 days)*						
0	19/116	1 Reference	.	59/156	1 Reference	.
1	63/189	1.59 (1.05, 2.43)	0.03	90/216	1.11 (0.87, 1.43)	0.40
2 or more	53/157	1.41 (0.90, 2.22)	0.14	89/193	1.13 (0.87, 1.47)	0.34
*Education (currently attending)*						
Vocational/Trade school	30/57	1.23 (0.90, 1.69)	0.20	40/67	1.14 (0.89, 1.47)	0.30
Gymnasium	33/119	0.89 (0.64, 1.26)	0.52	37/123	0.80 (0.58, 1.11)	0.18
Bachelor’s degree	9/78	0.40 (0.23, 0.68)	<.001	34/103	0.76 (0.56, 1.02)	0.07
Master’s degree	63/208	1 Reference	.	127/272	1 Reference	.
*Living Status*						
Left home	58/163	1 Reference	.	67/172	1 Reference	.
Live with parents	77/299	0.83 (0.63, 1.08)	0.17	171/393	1.09 (0.86, 1.39)	0.46
*BMI level*						
Underweight	3/14	0.61 (0.24, 1.59)	0.31	7/18	0.93 (0.51, 1.70)	0.82
Normal	87/347	1 Reference	.	174/434	1 Reference	.
Overweight	37/89	1.26 (0.93, 1.71)	0.13	44/96	1.05 (0.82, 1.35)	0.68
Obese	8/12	2.00 (1.27, 3.15)	0.003	13/17	1.57 (1.17, 2.11)	0.003
Non-modifiable variables						
*Season*						
Summer	11/140	1 Reference	.	41/170	1 Reference	.
Winter	124/322	3.84 (2.16, 6.85)	<.001	197/395	1.76 (1.30, 2.39)	<.001
*Ethnicity/Race*						
Caucasian	102/414	1 Reference	.	218/530	1 Reference	.
Other	33/48	2.16 (1.64, 2.84)	<.001	20/35	1.36 (0.99, 1.85)	0.05
*Sex*						
Women	45/226	1 Reference	.	113/294	1 Reference	.
Men	90/236	2.09 (1.52, 2.87)	<.001	125/271	1.31 (1.06, 1.61)	0.012

Alcohol intake of “5 or more” was associated with a lower RR = 0.68 (0.47, 0.90) for vitamin D deficiency compared to non-drinker, an increase of one unit of alcohol was associated with RR = 0.81 (0.68 - 0.97). There was no difference in RR for vitamin D insufficiency for different levels of alcohol intake (Table 2)

Having had one fast food meal in the preceding seven days was associated with a significantly higher RR = 1.59 (1.05, 2.43) for vitamin D deficiency compared to no fast food meals the last seven days, though the slope was not significant. Different levels of fast food intake had the same RR for vitamin D insufficiency.

Education at the Bachelor’s degree level had the lowest RR = 0.40 (0.23, 0.68) compared to a master degree for vitamin D deficiency, and for the length of education (currently attending); the slope for one-year of additional education was non-significant. For vitamin D insufficiency, the RR was equal among educational levels.

Living status did not influence RR for vitamin D insufficiency or deficiency.

BMI in the obese category had the highest RR = 2.00 (1.27, 3.15) for vitamin D deficiency compared to normal, and one unit extra BMI was associated with RR = 1.06 (1.02 - 1.10). For vitamin D insufficiency, the obese group had the highest RR = 1.57 (1.17, 2.11) compared to normal; however, one unit of extra BMI was associated with RR = 1.02 (0.99 - 1.04)

### Non-modifiable variables

**Fig. 2:**
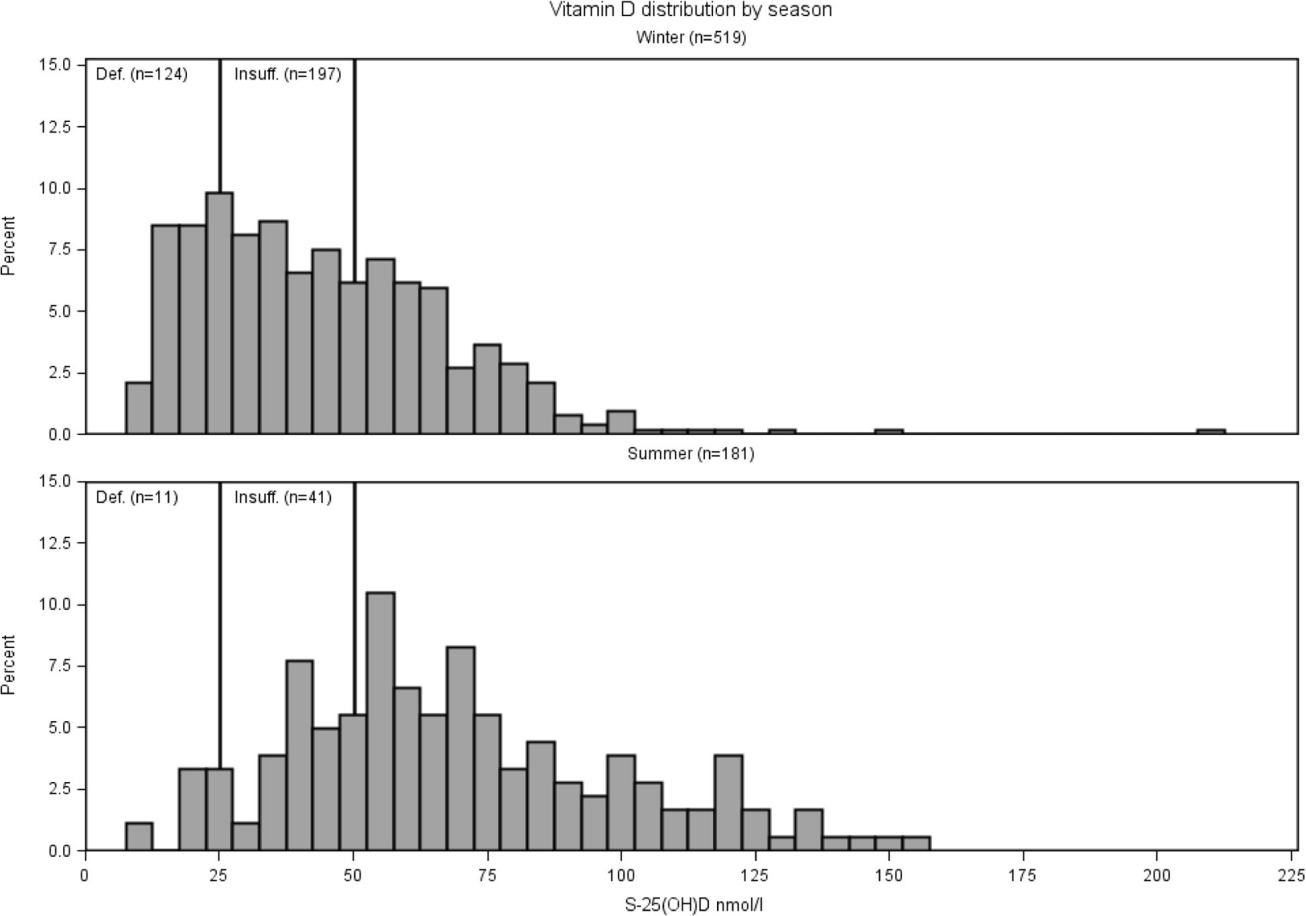
Vitamin D (S-25[OH]D) distribution by season. For each season, the number of participants with vitamin D deficiency (Def.), vitamin D insufficiency (Insuff.) and the total number is stated at the top of each figure

In the summer, the unadjusted mean for S-25[OH]D was 61.7 (37.7, 101) nmol/L; the winter unadjusted mean for S-25[OH]D was 38.3 (21.9, 66.8) nmol/L. The distributions of S-25[OH]D in summer and winter are illustrated in Fig. 2. For vitamin D deficiency in winter RR = 3.84 (2.16, 6.85) and for vitamin D insufficiency RR = 1.76 (1.30, 2.39)

Women had significantly lower RR compared to men; for vitamin D insufficiency RR = 0.74 (0.55, 0.997) and for deficiency RR = 0.48 (0.35, 0.66). Season and sex did not interact.

Non-Caucasian ethnicity had a significantly higher RR for vitamin D deficiency RR = 2.16 (1.64, 2.84) but not for vitamin D insufficiency RR = 1.36 (0.99, 1.85).

In our study population, participants with the lowest RR for vitamin D deficiency were Caucasian women with a Bachelor’s level education, normal BMI, physically active and non-smoking lifestyle, and alcohol intake of 5 or more in the preceding seven days.

## Discussion

Our cross-sectional, youth-based study shows that exercise, alcohol intake, BMI level, sex, ethnicity, tobacco use, and fast-food consumption are associated with risk for vitamin D deficiency and that exercise, season, BMI level and sex are associated with risk for vitamin D insufficiency.

We found that almost one-fifth of the youth included in this study suffered from vitamin D deficiency, and more than half had vitamin D insufficiency or worse. This observation is in line with existing observations from the same geographic area [29]. Theoretically, living in central Europe could decrease the risk of vitamin D deficiency/insufficiency due to increased exposure to sunlight; however, vitamin D insufficiency is also prevalent in other parts of Europe [31, 32].

Youth participating in outdoor sports are expected to have higher circulating S-25[OH]D and thus higher vitamin D status. In line with our expectations, we found increasing time spent exercise were associated with higher vitamin d status. This is consistent with results reported elsewhere [16, 21, 29, 33]. In those under 32 years of age, vitamin D is shown to affect positively muscles [34]. Exercise, gender or season did not interact, thus only strengthening the association between exercise and increased vitamin D status. This observation might be influenced by sun exposure via outdoor exercise. Exposure to sunlight is the major source of vitamin D, a variable we haven’t included due to the cross-sectional nature of the study. The effect of sunlight on the cutaneous vitamin D synthesis can be modified by sunscreen [35] and clothes [36]. People with dark skin tone have natural sun protection. Thus, longer sun exposure is necessary to make the same amount of vitamin D in the skin [37]. To our knowledge, this association between exercise and a vitamin D status has not previously been reported in young adults. Though sun exposure is a possible confounder and the association may simply be related to outdoor activities and general lifestyle.

Smokers had a significantly higher risk of vitamin D deficiency but not insufficiency; the suggested impact of smoking on vitamin D in this study is consistent with earlier studies [24, 29, 33, 38, 39]. A causative effect can not be excluded as metabolites of smoking (tetralones) might upset the vitamin D metabolism by inhibiting the CYP27A1 activity [40], CYP27A1 is found in the liver and takes part in the hydroxylation of vitamin D to 25-hydrozyvitamin D. However the effect of smoking cessation in healthy adults on 25[OH]D is not as clear as one would expect [41]. Smoking is only associated with the vitamin D deficiency, which is known to affect the bone mineralisation process; smoking is also associated with lower bone mineral density (BMD) in young men [42].

Alcohol intake was found to be positively related to decreased RR for vitamin D deficiency. No interaction was found between gender and alcohol consumption. Former studies in adults have described an apparently beneficial effect of alcohol [16, 23, 43]. No prospective studies on humans have been carried out with regards to alcohol administration and 25-hydroxyvitamin D. However in rats feed vitamin D_3_ and ethanol, for the ethanol group plasma 25[OH]D increased, and more vitamin D_3_ was converted to 25[OH]D and 25[OH]D was not incorporated into muscle and adipose tissue to the same extent as in control group only feed vitamin D_3_, it should be noticed that vitamin D binding protein was not measured [44]. The other possible contributor to altered 25[OH]D is vitamin D binding protein, but it doesn’t differ between alcoholics and non-alcoholics [45]. Whether the same effect of ethanol on 25[OH]D exists in humans too is unknown. Chronic alcoholism is associated with low 25[OH]D [46]. Others have reported a sex difference between the positive association between 25[OH]D and alcohol intake could [47], we did not find this. The possible benefits of moderate drinking and S-25[OH]D are of interest, but the uncertainties associated herewith does not allow the recommendation to increase alcohol consumption before prospective studies are carried out.

The ingestion of one fast food meal per week was associated with increased risk of vitamin D deficiency, but there was no significant trend. Only one study has investigated fast food intake and vitamin D deficiency and reported an inverse association between S-25[OH]D and fast-food consumption [48]. Overall, few data exists on dietary habits regarding processed food sold over the counter and vitamin D.

Analysis of education (currently attending) indicated that subjects studying for a Bachelor´s degree fared remarkably well both in terms of highest S-25[OH]D and the lowest risk of vitamin D insufficiency and deficiency. The impact of education on vitamin d status has been acknowledged in previous research, although only winter results have been reported [39]. It is known that obesity follows a socioeconomic gradient, and social class is linked to diet quality [17, 49]. Given this, we would expect an interaction between BMI levels and educational level. However, we did not find this in our analysis, and there was no significant interaction between educational level and fast food consumption. Because we only have an educational level (currently attending) as a marker of socioeconomic status, not economic data, we cannot rule out an impact of socioeconomic status on vitamin d status.

Gender had a striking effect on serum vitamin d status. We observed a close association between sex and the vitamin d status. In our study, there was a significantly higher prevalence of deficiency among men (24.9 %) than women (13.4 %), which is similar to studies reporting data from areas with the same latitude as Denmark [33]. Use of oral anti-contraceptives (i.e., oestrogen) could also play a role, as they are known to increase S-25[OH]D [50] by an increase in vitamin D binding protein [51], although the free index of the active vitamin D 1,25[OH]D does not change [51]. Studies performed in other regions [38] reported significantly different observations; regional variations in contraception could provide a partial explanation for the various observations [52].

We expected and observed was that ethnicity - “Caucasians” compared with “Non-Caucasians” - yielded a significant difference in S-25[OH]D status. Genetic differences in vitamin D binding protein across ethnicities could partially explain this difference [53].

The well-known seasonal fluctuation in S-25(OH)D concentration and the impact on the prevalence of insufficiency and deficiency were demonstrated as expected in this study. This is in accordance with previous results [29, 33, 38], and highlights the fact that the season needs to be taken into consideration especially when investigating other factors influencing vitamin d status and physiological variables affected by vitamin D [29, 38].

There is an ongoing debate related to the optimal concentration of S-25(OH)D. Some authors have recommended levels of S-25[OH]D 70–80 nmol/L reduce fracture risk [54]. Vitamin D supplementation has musculoskeletal benefits especially for age above 65 years [34, 55]. There are no consensus on optimal serum 25(OH)D concentrations for other health outcomes [56]. In the summer and winter, only 30.6 % and 6.9 %, respectively, had a vitamin d status above the recommended 75 nmol/L. The high rate of vitamin D deficiency could have severe health implications if not corrected. However, this is pure speculation, and there is currently no such evidence. Particular attention should be paid to the relatively small group of participants suffering severe vitamin D deficiency (S-25[OH]D < 12.5 nmol/L). In the summer, two of eleven non-Caucasians had severe vitamin D deficiency and in the winter, six out of 55 non-Caucasians had severe vitamin D deficiency. The group of non-Caucasians had a un-proportionally high prevalence of severe vitamin D deficiency, meaning that there is a significant group of youth facing high risks of rickets and osteomalacia when compared to the background population.

The three non-modifiable variables gender, season and race/ethnicity are best used in targeting high-risk groups (i.e., males of non-Caucasian origin especially in the winter) when designing trials of intervention. The two only modifiable variables that were consistent were time spent in exercise and BMI. There are several options to improve nutritional status for high-risk individuals. Recommendation of food rich in vitamin D such as fatty fish is one possibility. We didn’t have data on consumption of fish which is a primary food source of vitamin D [14], this is a limitation of the study. Even if we had such data, we wouldn’t know whether or not the high-risk groups would increase fish consumption if it was recommended. Information about food preferences and economic situation would be needed, so one doesn’t recommend high priced fish to a low-income group or a group who had no tradition of fish consumption. Two other options are vitamin D supplementation and food fortification both is viable but has implications too. We don’t have data on parathyroid hormone (PTH), but we would expect to see increased PTH in approximately on third of the subjects with vitamin D deficiency [7]. A recommendation of vitamin D supplementation would induce overtreatment. Secondly, we didn’t have data on vitamin D supplementation in this age group, and third we don’t know the preferences of the high-risk group with regards to supplementation. Food fortification has some the same problems as vitamin D supplementation. The lack of information on preferences for fish consumption and vitamin D supplementation in the high-risk groups make it difficult to estimate numbers needed to treat. With the current knowledge, it’s nearly impossible to implement such a strategy. The lowest risk for overtreatment with vitamin D supplementation would be in the winter and could be the first step in an intervention trial.

Low 25-hydroxyvitamin D has been associated with poor outcome for autoimmune diseases; type one diabetes [57], multiple sclerosis [58] and increased risk for metabolic syndrome [59], and hypertension [60]. This has led to a plethora of interventional trials. The intervention trials for type one diabetes [61], multiple sclerosis [62], metabolic syndrome [63] and hypertension [64] has been less than promising. This has not diminished the value of vitamin D as a predictor of outcome, but the lack of effect of supplementation could lead to believe that 25-hydroxyvitamin D is an innocent bystander and just marker of health status.

The definition of vitamin D deficiency forms the basis for the criteria for vitamin D supplementation. Though un-modifiable, season, gender and ethnicity all showed a high RR for vitamin D deficiency between groups. Therefore, emphasis should be placed on smoking, exercise and BMI as they are all modifiable and have the largest influence on RR for vitamin D deficiency.

### Strengths and limitations

This study has several strengths including a large sample of young adults consisting of men and women from different educational institutions living at the same latitude. Blood sampling took place during the summer as well as through wintertime. S-25[OH]D is a reliable marker of individual vitamin D status as it reflects vitamin D obtained from food sources and cutaneous synthesis, and it is not prone to diurnal variation.

On the other hand, there are potential limitations of this study. The cross-sectional study design does not allow for a temporal ordering of the associations between S-25[OH]D and various factors to be determined. We did not assess dietary or fatty fish intake, recent holidays in a sunny climate or outdoor time spent exercising all of which could be potential confounders.

Researchers have struggled to recruit young adults, and some methods of communication have proven more valuable than others [65]. the questionnaire was developed specifically for this study, with the aim of speed and convenience for the participant to ease recruitment. This approach was a tradeoff between easy recruitment, speed, convenience for participants and more information for the study.

Another limitation is the inclusion only of participants from educational institutions. Young adults who were not attending education were left out, introducing a possible bias. Education after primary school is not compulsory in Denmark. In 2012, 11,183 out of 70,840 18-year-old adults did not pursue further education [66]. Differences in S-25[OH]D likely exist between individuals who do and those who do not attend education after primary school. Therefore, it is likely that our data underestimates the real prevalence of vitamin D insufficiency and deficiency in the Copenhagen area.

## Conclusion

In conclusion, young adults in the Copenhagen area have a poor vitamin D status compared with the official recommendation by Danish authorities. Of particular concern is the group of vitamin D-deficient patients (6.6 % during the winter and 1.1 % during the summer) that suffers from severe vitamin D deficiency. The group at highest risk for vitamin D deficiency consisted mostly of smoking, non-drinking men with high BMI and low levels of exercise. Fast food consumption was inconsistently associated with vitamin D status.

Overall, the vitamin D status of the investigated population is poor. We suggest that the importance of vitamin D intake should be systematically addressed with a focus on the groups at highest risk of severe insufficiency; with the modifiable lifestyle factors in mind. Second, the dynamics of vitamin D status and exercise requires further exploration. As modifiable risk factors smoking and exercise could have significant potential for both, directly and indirectly, reducing the risk of vitamin D deficiency and the long-term consequences of vitamin D deficiency, including falls, osteoporosis, and fractures.

## Consent

All participants included in the study signed an informed consent. Participants answered a self-administered questionnaire about weight, height, smoking, alcohol, exercise habits, and fast food intake. In return, participants were provided with an interpreted answer of their S-25[OH]D and compensated with a gift card valued 6.71€ (exchange rate 1. of May 2012).

## Ethics

The regional ethics committee (Research Ethics Committee of the Capital Region of Denmark) approved this study, ref. no. H-1-2012-023.

## Consent for publication

Not applicable.

## Abbreviations

1,25-[OH]D: 1,25-dihydroxyvitamin D
25[OH]D: 25-hydroxyvitamin D
BMD: bone mineral density
BMI: body mass index
cholecalciferol: vitamin D_3_
ergocalciferol: Vitamin D_2_
IQR: interquartile range
PBM: peak bone mass
PTH: parathyroid hormone
RR: relative risk
S-: serum
Summer: 15 April -14 October
Vitamin D deficiency: S-25[OH]D < 25 nmol/L
Vitamin D insufficiency: 25 nmol/L ≤ S-25[OH]D ≤ 50 nmol/L
Vitamin D sufficiency: S-25[OH]D > 50 nmol/L
Winter: 15 October to 14 April

## References

[1] Seamans KM, Cashman KD (2009). Existing and potentially novel functional markers of vitamin D status: a systematic review. AmJClinNutr.

[2] Lanske B, Densmore MJ, Erben RG (2014). Vitamin D endocrine system and osteocytes. BonekeyRep.

[3] Wren TA, Kalkwarf HJ, Zemel BS, Lappe JM, Oberfield S, Shepherd JA, Winer KK, Gilsanz V. Longitudinal tracking of dual-energy X-ray absorptiometry bone measures over 6 years in children and adolescents: persistence of Low bone mass to maturity. J Pediatr. 2014. 10.1016/j.jpeds.2013.12.040 PMC4035430 24485819

[4] Bischoff-Ferrari HA, Dawson-Hughes B, Staehelin HB, Orav JE, Stuck AE, Theiler R, Wong JB, Egli A, Kiel DP. Fall prevention with supplemental and active forms of vitamin D: a meta-analysis of randomised controlled trials. BMJ. 2009;339:b3692. 10.1136/bmj.b3692 PMC2755728 19797342

[5] Cameron ID, Murray GR, Gillespie LD, Robertson MC, Hill KD, Cumming RG (2010). Interventions for preventing falls in older people in nursing care facilities and hospitals. Cochrane Database Syst Rev.

[6] Heaney RP (2003). Long-latency deficiency disease: insights from calcium and vitamin D. Am J Clin Nutr.

[7] Lips P (2004). Which circulating level of 25-hydroxyvitamin D is appropriate?. J Steroid Biochem Mol Biol.

[8] Holick MF (2004). Sunlight and vitamin D for bone health and prevention of autoimmune diseases, cancers, and cardiovascular disease. Am J Clin Nutr.

[9] Ebeling PR (2014). Vitamin D and bone health: epidemiologic studies. Bonekey Rep.

[10] Stagi S, Cavalli L, Iurato C, Seminara S, Brandi ML, de Martino M (2013). Bone metabolism in children and adolescents: main characteristics of the determinants of peak bone mass. Clin Cases Miner Bone Metab.

[11] Hogstrom M, Nordstrom A, Nordstrom P (2006). Relationship between vitamin D metabolites and bone mineral density in young males: a cross-sectional and longitudinal study. CalcifTissue Int.

[12] Kristinsson JO, Valdimarsson O, Sigurdsson G, Franzson L, Olafsson I, Steingrimsdottir L (1998). Serum 25-hydroxyvitamin D levels and bone mineral density in 16-20 years-old girls: lack of association. JInternMed.

[13] Webb AR, Kline L, Holick MF (1988). Influence of season and latitude on the cutaneous synthesis of vitamin D3: exposure to winter sunlight in Boston and Edmonton will not promote vitamin D3 synthesis in human skin. J Clin Endocrinol Metab.

[14] Burgaz A, Akesson A, Oster A, Michaelsson K, Wolk A (2007). Associations of diet, supplement use, and ultraviolet B radiation exposure with vitamin D status in Swedish women during winter. Am J Clin Nutr.

[15] Cheng S, Massaro JM, Fox CS, Larson MG, Keyes MJ, McCabe EL, Robins SJ, O'Donnell CJ, Hoffmann U, Jacques PF, et al. Adiposity, cardiometabolic risk, and vitamin D status: the Framingham Heart Study. Diabetes. 2010;59(1):242–8. 10.2337/db09-1011 PMC2797928 19833894

[16] Skaaby T, Husemoen LL, Thuesen BH, Pisinger C, Hannemann A, Jorgensen T, Linneberg A. Longitudinal associations between lifestyle and vitamin D: a general population study with repeated vitamin D measurements. Endocrine. 2015. 10.1007/s12020-015-0641-7 26024976

[17] Drewnowski A (2009). Obesity, diets, and social inequalities. Nutr Rev.

[18] Groth MV, Fagt S, Brondsted L (2001). Social determinants of dietary habits in Denmark. Eur J Clin Nutr.

[19] Jarman M, Lawrence W, Ntani G, Tinati T, Pease A, Black C, Baird J, Barker M, Group SIHS. Low levels of food involvement and negative affect reduce the quality of diet in women of lower educational attainment. J Hum Nutr Diet. 2012;25(5):444–52. 10.1111/j.1365-277X.2012.01250.x 22515167

[20] Jaaskelainen T, Knekt P, Marniemi J, Sares-Jaske L, Mannisto S, Heliovaara M, Jarvinen R. Vitamin D status is associated with sociodemographic factors, lifestyle and metabolic health. Eur J Nutr. 2013;52(2):513–25. 10.1007/s00394-012-0354-0 22538929

[21] Wanner M, Richard A, Martin B, Linseisen J, Rohrmann S (2015). Associations between objective and self-reported physical activity and vitamin D serum levels in the US population. Cancer Causes Control.

[22] Constantini NW, Dubnov-Raz G, Chodick G, Rozen GS, Giladi A, Ish-Shalom S (2010). Physical activity and bone mineral density in adolescents with vitamin D deficiency. Med Sci Sports Exerc.

[23] Larose TL, Chen Y, Camargo CA, Langhammer A, Romundstad P, Mai XM (2014). Factors associated with vitamin D deficiency in a Norwegian population: the HUNT Study. J Epidemiol Community Health.

[24] Kassi EN, Stavropoulos S, Kokkoris P, Galanos A, Moutsatsou P, Dimas C, Papatheodorou A, Zafeiris C, G L. Smoking is a significant determinant of low serum vitamin D in young and middle-aged healthy males. Hormones(Athens). 2014;14(2):245–50. 10.14310/horm.2002.1521 25402376

[25] Hossein-nezhad A, Holick MF (2013). Vitamin D for health: a global perspective. Mayo Clin Proc.

[26] Bouillon R, Van Schoor NM, Gielen E, Boonen S, Mathieu C, Vanderschueren D, Lips P. Optimal vitamin D status: a critical analysis on the basis of evidence-based medicine. J Clin Endocrinol Metab. 2013;98(8):E1283–304. 10.1210/jc.2013-1195 23922354

[27] Christensen LM, Kørup K, Trolle E, Matthiessen J, Fagt S (2007). Børn og unges måltidsvaner 2000-2004.

[28] Durup D, Jorgensen HL, Christensen J, Schwarz P, Heegaard AM, Lind B (2012). A reverse J-shaped association of all-cause mortality with serum 25-hydroxyvitamin D in general practice: the CopD study. JClinEndocrinolMetab.

[29] Thuesen B, Husemoen L, Fenger M, Jakobsen J, Schwarz P, Toft U, Ovesen L, Jorgensen T, Linneberg A. Determinants of vitamin D status in a general population of Danish adults. Bone. 2012;50(3):605–10. 10.1016/j.bone.2011.12.016 22227435

[30] Zou G (2004). A modified poisson regression approach to prospective studies with binary data. Am J Epidemiol.

[31] Gonzalez-Gross M, Valtuena J, Breidenassel C, Moreno LA, Ferrari M, Kersting M, De HS, Gottrand F, Azzini E, Widhalm K, et al. Vitamin D status among adolescents in Europe: the Healthy Lifestyle in Europe by Nutrition in Adolescence study. BrJNutr. 2012;107(5):755–64. 10.1017/S0007114511003527 21846429

[32] McKenna MJ (1992). Differences in vitamin D status between countries in young adults and the elderly. Am J Med.

[33] Oberg J, Jorde R, Almas B, Emaus N, Grimnes G (2014). Vitamin D deficiency and lifestyle risk factors in a Norwegian adolescent population. Scand J Public Health.

[34] Tomlinson PB, Joseph C, Angioi M. Effects of vitamin D supplementation on upper and lower body muscle strength levels in healthy individuals. A systematic review with meta-analysis. J SciMed Sport 2014;18(5):575-80. 10.1016/j.jsams.2014.07.022 25156880

[35] Matsuoka LY, Ide L, Wortsman J, MacLaughlin JA, Holick MF (1987). Sunscreens suppress cutaneous vitamin D3 synthesis. J Clin Endocrinol Metab.

[36] Hatun S, Islam O, Cizmecioglu F, Kara B, Babaoglu K, Berk F, Gokalp AS. Subclinical vitamin D deficiency is increased in adolescent girls who wear concealing clothing. JNutr. 2005;135(2):218–22. 10.1093/jn/135.2.218 15671216

[37] Sawicki CM, Van Rompay MI, Au LE, Gordon CM, Sacheck JM (2016). Sun-exposed skin color is associated with changes in serum 25-hydroxyvitamin D in racially/ethnically diverse children. J Nutr.

[38] Gill TK, Hill CL, Shanahan EM, Taylor AW, Appleton SL, Grant JF, Shi Z, Grande ED, Price K, Adams RJ. Vitamin D levels in an Australian population. BMC Public Health. 2014;14(1):1001. 10.1186/1471-2458-14-1001 PMC4194387 25256413

[39] Shinkov A, Borissova AM, Dakovska L, Vlahov J, Kassabova L, Svinarov D. Winter 25-hydroxyvitamin D levels in young urban adults are affected by smoking, body mass index and educational level. Eur J Clin Nutr. 2014. 10.1038/ejcn.2014.163 25117996

[40] Aboraia AS, Makowski B, Bahja A, Prosser D, Brancale A, Jones G, Simons C. Synthesis and CYP24A1 inhibitory activity of (E)-2-(2-substituted benzylidene)- and 2-(2-substituted benzyl)-6-methoxy-tetralones. Eur J Med Chem. 2010;45(10):4427–34. 10.1016/j.ejmech.2010.07.001 20655626

[41] Supervia A, Nogues X, Enjuanes A, Vila J, Mellibovsky L, Serrano S, Aubia J, Diez-Perez A. Effect of smoking and smoking cessation on bone mass, bone remodeling, vitamin D, PTH and sex hormones. J Musculoskelet Neuronal Interact. 2006;6(3):234–41. 17142943

[42] Valimaki MJ, Karkkainen M, Lamberg-Allardt C, Laitinen K, Alhava E, Heikkinen J, Impivaara O, Makela P, Palmgren J, Seppanen R, et al. Exercise, smoking, and calcium intake during adolescence and early adulthood as determinants of peak bone mass. Cardiovascular Risk in Young Finns Study Group. BMJ. 1994;309(6949):230–5. 10.1136/bmj.309.6949.230 PMC2540782 8069139

[43] Nakamura K, Kitamura K, Takachi R, Saito T, Kobayashi R, Oshiki R, Watanabe Y, Tsugane S, Sasaki A, Yamazaki O. Impact of demographic, environmental, and lifestyle factors on vitamin D sufficiency in 9084 Japanese adults. Bone. 2015. 10.1016/j.bone.2014.12.064 25576673

[44] Gascon-Barre M (1982). Interrelationships between vitamin D3 and 25-hydroxyvitamin D3 during chronic ethanol administration in the rat. Metabolism.

[45] Bjorneboe GE, Bjorneboe A, Johnsen J, Skylv N, Oftebro H, Gautvik KM, Hoiseth A, Morland J, Drevon CA. Calcium status and calcium-regulating hormones in alcoholics. Alcohol Clin Exp Res. 1988;12(2):229–32. 10.1111/j.1530-0277.1988.tb00185.x 2837104

[46] Laitinen K, Valimaki M, Lamberg-Allardt C, Kivisaari L, Lalla M, Karkkainen M, Ylikahri R. Deranged vitamin D metabolism but normal bone mineral density in Finnish noncirrhotic male alcoholics. Alcohol ClinExpRes. 1990;14(4):551–6. 10.1111/j.1530-0277.1990.tb01198.x 2221282

[47] Lee K (2012). Sex-specific relationships between alcohol consumption and vitamin D levels: The Korea National Health and Nutrition Examination Survey 2009. Nut Res Pract.

[48] Muhairi SJ, Mehairi AE, Khouri AA, Naqbi MM, Maskari FA, Al KJ, Al Dhaheri AS, Nagelkerke N, Shah SM. Vitamin D deficiency among healthy adolescents in Al Ain, United Arab Emirates. BMC Public Health. 2013;13:33. 10.1186/1471-2458-13-33 PMC3610121 23311702

[49] Darmon N, Drewnowski A (2008). Does social class predict diet quality?. Am J Clin Nutr.

[50] Harris SS, Dawson-Hughes B (1998). The association of oral contraceptive use with plasma 25-hydroxyvitamin D levels. J Am Coll Nutr.

[51] ten Bolscher M, de Valk-de Roo GW, Barto R, van der Vijgh WJ, Netelenbos JC (1999). Oestrogen has no short-term effect on intestinal strontium absorption in healthy postmenopausal women. Clin Endocrinol (Oxf).

[52] de Irala J, Osorio A, Carlos S, Lopez-del Burgo C (2011). Choice of birth control methods among European women and the role of partners and providers. Contraception.

[53] Powe CE, Evans MK, Wenger J, Zonderman AB, Berg AH, Nalls M, Tamez H, Zhang D, Bhan I, Karumanchi SA, et al. Vitamin D-binding protein and vitamin D status of black Americans and white Americans. N EnglJ Med. 2013;369(21):1991–2000. 10.1056/NEJMoa1306357 PMC4030388 24256378

[54] Dawson-Hughes B, Heaney R, Holick M, Lips P, Meunier P, Vieth R (2005). Estimates of optimal vitamin D status. Osteoporos Int.

[55] Beaudart C, Buckinx F, Rabenda V, Gillain S, Cavalier E, Slomian J, Petermans J, Reginster JY, Bruyere O. The effects of vitamin D on skeletal muscle strength, muscle mass, and muscle power: a systematic review and meta-analysis of randomized controlled trials. J Clin Endocrinol Metab. 2014;99(11):4336–45. 10.1210/jc.2014-1742 25033068

[56] Romagnoli E, Carnevale V, Biondi P, Minisola S (2014). Vitamin D supplementation: when and how?. J Endocrinol Investig.

[57] Feng R, Li Y, Li G, Li Z, Zhang Y, Li Q, Sun C. Lower serum 25 (OH) D concentrations in type 1 diabetes: a meta-analysis. Diabetes Res Clin Pract. 2015;108(3):e71–5. 10.1016/j.diabres.2014.12.008 25836943

[58] Alharbi FM (2015). Update in vitamin D and multiple sclerosis. Neurosciences (Riyadh).

[59] Ju SY, Jeong HS, Kim do H (2014). Blood vitamin D status and metabolic syndrome in the general adult population: a dose-response meta-analysis. J Clin Endocrinol Metab.

[60] Jorde R, Figenschau Y, Emaus N, Hutchinson M, Grimnes G (2010). Serum 25-hydroxyvitamin D levels are strongly related to systolic blood pressure but do not predict future hypertension. Hypertension.

[61] Dong JY, Zhang WG, Chen JJ, Zhang ZL, Han SF, Qin LQ (2013). Vitamin D intake and risk of type 1 diabetes: a meta-analysis of observational studies. Nutrients.

[62] Kampman MT, Steffensen LH, Mellgren SI, Jorgensen L (2012). Effect of vitamin D3 supplementation on relapses, disease progression, and measures of function in persons with multiple sclerosis: exploratory outcomes from a double-blind randomised controlled trial. Mult Scler.

[63] Gulseth HL, Gjelstad IM, Birkeland KI, Drevon CA (2013). Vitamin D and the metabolic syndrome. Curr Vasc Pharmacol.

[64] Beveridge LA, Struthers AD, Khan F, Jorde R, Scragg R, Macdonald HM, Alvarez JA, Boxer RS, Dalbeni A, Gepner AD, et al. Effect of vitamin D supplementation on blood pressure: a systematic review and meta-analysis incorporating individual patient data. JAMA Intern Med. 2015;175(5):745–54. 10.1001/jamainternmed.2015.0237 PMC5966296 25775274

[65] Moe SG, Lytle LA, Nanney MS, Linde JA, Laska MN (2016). Recruiting and retaining young adults in a weight gain prevention trial: Lessons learned from the CHOICES study. Clin Trials.

[66] Sørensen S.15-29 åriges uddannelser efter område, herkomst, højeste fuldførte uddannelse, igangværende uddannelse, alder og køn 2014 [http://www.statistikbanken.dk/KRHFU3 Accessed 10/30/2014 2014]. Accessed 19 June 2013

