# Erratum to: determinants of vitamin D status in young adults: influence of lifestyle, sociodemographic and anthropometric factors

**DOI:** 10.1186/s12889-017-4559-2

**Published:** 2017-07-12

**Authors:** Rune Tønnesen, Peter Hambak Hovind, Lars Thorbjørn Jensen, Peter Schwarz

**Affiliations:** 1 Department of Clinical Physiology and Nuclear Medicine, Rigshospitalet, Nordre Ringvej 57, 2600 Copenhagen, Glostrup, Denmark; 2 Department of Endocrinology PE and Research Centre of Ageing and Osteoporosis, Rigshospitalet, Copenhagen, Denmark; 3 Department of Clinical Physiology and Nuclear Medicine, University Hospital of Herlev, Copenhagen, Denmark; 4 Faculty of Health Sciences, University of Copenhagen, Copenhagen, Denmark

## Erratum

After publication of the article [1] it was brought to our attention that there are several typological errors in Table 1. ‘Height’ should be read in *cm* rather than *m* and there are significant errors in the reported ages of the subjects. The corrected version of the table can be found below.

**Table 1:** Characteristics of participants

Variables	Women (n=339)	Men (n=361)
Age (year)	22.0 ± 2.2	21.6 ± 2.3
Height (cm)	167.9 ± 6.6	182.8 ± 7.1
Weight (kg)	63.8 ± 10.7	79.5 ± 11.6
BMI (kg/m^2^)	22.6 ± 3.3	23.8 ± 3.0
S-25[OH]D* (nmol/L)	53 (35 – 69)	40 (25 - 63)
*Season*		
Summer (%)	26.0	25.8
Winter (%)	74.0	74.2
*Ethnicity/Race*		
Caucasian (%)	88.8	91.7
Other (%)	11.2	8.3

Normally distributed data are expressed as the mean ± SD. *Skewed distributions are expressed as median (IQR)
